# Sentinels in Salmon Aquaculture: Heart Rates Across Seasons and During Crowding Events

**DOI:** 10.3389/fphys.2021.755659

**Published:** 2021-11-26

**Authors:** Fletcher Warren-Myers, Malthe Hvas, Tone Vågseth, Tim Dempster, Frode Oppedal

**Affiliations:** ^1^Sustainable Aquaculture Laboratory – Temperate and Tropical (SALTT), School of Biosciences, University of Melbourne, Melbourne, VIC, Australia; ^2^Institute of Marine Research, Animal Welfare Group, Matre, Norway

**Keywords:** data storage tags, diurnal rhythm, fish welfare, stress, sea cage

## Abstract

Advances in tag technology now make it possible to monitor the behavior of small groups of individual fish as bioindicators of population wellbeing in commercial aquaculture settings. For example, tags may detect unusual patterns in fish heart rate, which could serve as an early indicator of whether fish health or welfare is becoming compromised. Here, we investigated the use of commercially available heart rate biologgers implanted into 24 Atlantic salmon weighing 3.6 ± 0.8 kg (mean ± SD) to monitor fish over 5 months in a standard 12 m × 12 m square sea cage containing ∼6,000 conspecifics. Post tagging, fish established a diurnal heart rate rhythm within 24 h, which stabilized after 4 days. Whilst the registered tagged fish mortality over the trial period was 0%, only 75% of tagged fish were recaptured at harvest, resulting in an unexplained tag loss rate of 25%. After 5 months, tagged fish were approximately 20% lighter and 8% shorter, but of the similar condition when compared to untagged fish. Distinct diurnal heart rate patterns were observed and changed with seasonal day length of natural illumination. Fish exhibited lower heart rates at night [winter 39 ± 0.2 beats per min (bpm), spring 37 ± 0.2 bpm, summer 43 ± 0.3 bpm, mean ± SE] than during the day (winter 50 ± 0.3 bpm, spring 48 ± 0.2 bpm, summer 49 ± 0.2 bpm) with the difference between night and day heart rates near half during the summer (6 bpm) compared to winter and spring (both 11 bpm). When fish experienced moderate and severe crowding events in early summer, the highest hourly heart rates reached 60 ± 2.5 bpm and 72 ± 2.4 bpm, respectively, on the day of crowding. Here, if the negative sublethal effects on fish that carry tags (e.g., growth rate) can be substantially reduced, the ability to monitor diurnal heart rate patterns across seasons and detect changes during crowding events, and using heart rate biologgers could be a useful warning mechanism for detecting sudden changes in fish behavior in sea cages.

## Introduction

The use of sentinel animals as biomonitoring indicators of population health (e.g., Pert et al., 2014; Andrewartha et al., 2015) has a huge potential for the farm production systems that contain thousands of individuals. Salmon aquaculture is a prime candidate for using sentinels as it is not possible to continually monitor the health and welfare of every individual fish (Føre et al., 2018a). Creating sentinels by adding data storage tags or acoustic tags to a few select fish within a sea cage (e.g., Cubitt et al., 2005; Føre et al., 2017) can enable the direct supply of information from individual fish to the fish farmer, and may inform farmers of the preliminary warning signs, indicating that the cage environment and/or the welfare of fish within the cage is starting to deteriorate. For example, in Atlantic salmon farmed in sea cages, deviating swimming behavior toward the surface of moribund individuals (Wright et al., 2019), hypoxia experience (Solstorm et al., 2018), high-temperature experiences (Johansson et al., 2009; Stehfest et al., 2017), and high activity levels occurs during crowding and lice treatment (Føre et al., 2018b).

Of particularly promise as a tool for bio sentinels as welfare indicators in aquaculture is the use of heart rate biologgers to monitor fish heart rates, which has shown to provide unique insights into the physiological and behavioral state of fish over time (Cooke et al., 2016; Prystay et al., 2017; Brijs et al., 2018, 2019a; Hvas et al., 2020a). For salmonids, heart rates are correlated with swimming activity and metabolic rates, and are influenced by temperature, hypoxia, and any acute or chronic stressor (Farrell, 2002; Brijs et al., 2018, 2019a; Hvas et al., 2020b; Svendsen et al., 2021). Moreover, Atlantic salmon normally display a strong diurnal heart rate rhythm, with lower heart rates at night and higher heart rates during the day (Hjelmstedt et al., 2020; Svendsen et al., 2021). A disruption of this diurnal rhythm has been suggested as a strong indicator of stress, whilst the re-establishment of a diurnal heart rate pattern reflects the required time for recovery (e.g., Brijs et al., 2018). In addition, continuously elevated heart rates above a *prior* established diurnal pattern may indicate that a fish is stressed or becoming chronically stressed (e.g., Hvas et al., 2020b).

To date, heart rate biologgers have been tested in controlled laboratory environments over periods of up to 13 weeks (Brijs et al., 2019b; Hvas et al., 2020a, b) and in sea cages from a few weeks (Brijs et al., 2018, 2019a) to 2 months during the summer (Svendsen et al., 2021). Whilst these studies have provided the foundation for monitoring heart rates in farmed fish, they have not yet demonstrated whether it is feasible or practical to use heart rate biologgers in sentinel fish as a long-term monitoring tool across multiple seasons in commercial sea cages. For example, it is still untested as to whether fish tagged with heart rate biologgers will have poorer long-term growth effects due to being tagged; whether heart rate biologgers will continue to read correctly as fish grow markedly larger; and how heart rates of tagged salmon in sea cages are compared to that of tagged fish in the laboratory environment, in terms of seasonal diurnal patterns and response to crowding or stress events. Here, we test the use of heart rate biologgers surgically implanted into Atlantic salmon to monitor their heart rate levels in a standard commercial sea cage over the winter–spring–summer period. We aimed to determine: (1) whether fish welfare measures such as survival, growth, and condition were similar in tagged and untagged fish in a sea cage environment; (2) what are the typical diurnal heart rate rhythms of farmed fish in a sea cage environment during winter, spring, and summer; and (3) how effective heart rate biologgers are at detecting fish stress levels and rates of recovery during crowding events in a sea cage (Noble et al., 2018).

## Materials and Methods

### Animal Ethics

All fish handling and surgery were made in compliance with the Norwegian animal welfare act and were approved by the Norwegian Animal Research Authority (permit no. 19629).

### Sea Cage

Monitoring of salmon in the final 5 months of sea-caged production was conducted from February to June 2020 at the Institute of Marine Research facility at Smørdalen, Masfjorden, Norway (60°N). Fish were put to sea in June 2019 and experienced standard husbandry conditions throughout. The sea cage used was 12 m × 12 m × 12 m deep (1,728 m^3^) and contained 6,031 Atlantic salmon (Aquagen, Norway). Fish were fed (spirit S 75-50A3, Spirit 4.5 mm Nutra and Premium 4.5, 7, 1,200 pellet size according to fish size, Skretting, Norway) to satiation daily *via* an automatic feeding system over a 5-month trial. Feed delivery times were between 08:45 and 15:00 in winter darkness and 05:30 to 15:00 from May 2020 onward. Feeding was stopped on the 14th of June 2020 in preparation for the final sampling and slaughter. Any fish mortalities occurring during the trial period were accounted for by removing them *via* a suction pipe (LiftUp, Eikelandsosen, Norway, liftup.no) connected to the bottom of the cage daily.

### Crowding Events

In salmon aquaculture, one of the key handling actives, which can result in fish kills, is the crowding of fish in sea cages, which has resulted in a crowding scale being developed to evaluate fish welfare and stress levels during crowding events (Noble et al., 2018). The crowding scale (1–5) evaluates the degree of crowding based mainly on observations of fish behavior at the surface. Briefly, level 1 is considered as the goal standard, meaning low stress for the fish with no vigorous activity observed on the surface; level 2 is acceptable, with the occurrence of normal swim behavior and some dorsal fins on the surface; level 3 is undesirable, with excited random directional swim behavior, and > 20 fins on the surface; level 4 is unacceptable overcrowding, with many fish stuck against the crowd net, numerous dorsal fins, and white sides visible on the surface, plus the occasional lethargic fish. Finally, level 5 is considered as extreme crowding, where there is panic in the population, fish are exhausted, with many floating on their side, and the risk of a high fish kill event is likely. During this trial, in the weeks leading up to harvest, we enacted the equivalent of a level 2 (acceptable) and level 3 (undesirable) crowding event, to determine if a measurable heart rate response can be used as an indicator of fish stress levels during crowding.

### Environment

**FIGURE 1:**
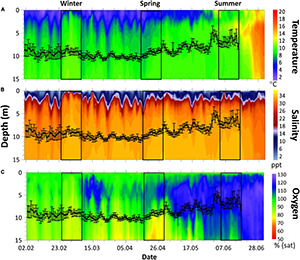
Environmental contour plot from the 2nd February to the 2nd of July. About 0–15 m depth of water temperature **(A)**, salinity **(B)**, and oxygen saturation **(C)**. Black line represents 24 h mean swimming depth (± 1 SD) of all fish in the cage estimated from the cage echo sounder data.

The marine environment in the fjord was monitored by an automatic profiling buoy (APB5, SAIV A/S, Laksevåg, Norway) profiling temperature, salinity, and oxygen daily from 0 to 15 m depth. Swimming depth distributions of fish in the cage that contained the fish tagged with the bio-loggers were continuously recorded throughout the experimental period using a PC-based echo integration system (see Warren-Myers et al., 2021, and the references therein) to give an indication of the environmental conditions that > 50% of all fish in the cage experienced during the trial (Figure 1).

### Heart Rate Biologger Tags

Heart rate biologgers (DST centi-HRT, dimensions: 46 mm × 15 mm, weight in air 19 g^1^) were implanted into 24 fish in February 2020 (12 on the 7th of February and 12 more on the 11th of February). Prior to implanting, the loggers and all surgical equipment were sterilized in a pure alcohol solution. To implant loggers, a random group of fish were trapped in the upper surface waters of the sea cage using a casting net (inside the volume of the net ∼ 4 m × 4 m × 3 m = 48 m^3^) pulled up from 5 to 7 m depth during hand feeding and then one at a time fish were transferred with a large dip net to an ambient seawater bath (Temp ∼8°C) that contained 150 mg L^–1^ of Finquel MS-222 (Tricaine Methanesulfonate) to anesthetize the fish. Once fully anesthetized (∼5–6 min), fish were then placed ventral side up on a cradle with anesthesia continued by gravity feeding recirculated seawater containing 75 mg L^–1^ Finquel MS-222 *via* a tube over the gills. A 3–4 cm incision was made into the abdominal cavity in the center of the ventral plane, just posterior to the pectoral fins. A heart rate logger was then pushed into the abdominal cavity flat end first toward the pericardium with the two electrodes orientated ventrally toward the abdominal muscles of the body wall (Brijs et al., 2018). The logger was then held in place with a short length of non-absorbable suture (4.0 USP RESOLON^®^ Blue) attached to the rounded end of the logger using a single stich that also closed the anterior end of the incision. Two to three further stiches were used to close the remainder of the incision. The wound was then gently cleaned of any blood or fluid with tissue wipes before a thin layer of Histoacryl^®^ was applied to the area to seal the wound. Fish were externally tagged near the dorsal fin with a yellow Floy tag (Hallprint^®^) and then measured, weighed, and transferred to a 1,000-L holding tank containing flow through ambient seawater until they were awake and reached equilibrium (10–15 min) before being returned to the sea cage. The tagging procedure from the time when fish were first initially placed in an anesthetic bath, tagged, and then moved to the recovery tank took approximately 13–15 min.

### Fish Condition and Growth

A random sample of 30 fish were collected from the sea cage on the 3rd of February 2020 and the 22nd of June 2020 for an initial and a final cage estimate of untagged fish length, weight, and condition factor. For tagged fish, 12 fish were captured, measured (fork length), weighed, and tagged on the 7th of February and the other 12 on the 11th of February, making a total of 24 tagged individuals. During the final sampling on the 22nd of June, 12 identifiable (*via* an external Floy tag) tagged fish were recaptured using a dip net, killed by an overdose of anesthetic, measured (fork length), and weighed and had their tags retrieved. Remaining identifiable tagged fish (six) were collected from the slaughter boat at harvest on the 2nd of July.

### DST Centi-HRT Data Collection

Fish heart rate and temperature data were logged continuously from the 7th of February 2020 to the 3rd of July 2020. For this period, heart rate biologgers were programmed using the Mercury v5.20 software to record eight consecutive heartbeat measurements every hour, with a measuring period of 15 s at 80 Hz. The same sampling period was used for temperature. Post-surgery recovery was assessed using the first 12 days of hourly heart rate data from the17 recaptured tagged fish. To compare seasonal changes in heart rate and diurnal rhythms, individual hourly heart rate and temperature data measurements from these 17 fish are selected from the 24th of February to the 8th of March as a snapshot of winter, the 17th to the 30th of April as a snapshot of spring, and the 2nd to the 15th of June as a snapshot of early summer. These 14-day periods were chosen as they aligned with known times when standard work activity occurred at the farm site.

To assess the fish heart rate response to crowding events, on the 16th of June the bottom of the net cage was lifted for 3 h between 10:00 and 13:00 to simulate a level 2 crowding event. Hourly heart rate measurements from the recaptured tagged fish (*n* = 17) for the day before crowding (the 15th of June) during (the 16th of June) and after (the 17th of June) were used to assess the effect of the level 2 crowding. During the final sampling on the 22nd of June, the net cage was lifted for 5 h and all fish crowded whilst farm technicians actively select the sampled fish from the cage with dip nets, equating to a level 3 crowding event for 80% of the time. For a graphical representation of the level 3 crowding on the 22nd of June, all hourly heart rate and temperature measurements from the last 16 days leading up to harvest (the 17th of June to the 2nd of July) were selected using the remaining six tagged fish recaptured on the slaughter boat on the 2nd of July. Hourly heart rate measurements from the day before crowding (the 21st of June), on the day of crowding (the 22nd of June), and the day after crowding (the 23rd of June) are used to assess the effect of level 3 crowding.

### Data Cleaning

Data from the heart rate biologgers were downloaded using the proprietary base station and software (Mercury v5.20, Star Oddi, Garðabaer, Iceland). Due to only having eight measurements per hour for the measures of heart rate and temperature, QI_0_ and QI_1_ values were used to avoid the fragmentation of the data set. QI values give an indication of data quality with QI_0_ values considered to be the most accurate heart rate measurements, QI_1_ the next best, QI_2_ fair, and QI_3_ poor. When combining QI_0_ and QI_1_ values, if multiple heart rate measurements are averaged, the margin of error will quickly decline to < 3 beats per min (bpm) (Brijs et al., 2019b). Across the 17 fish with useable data, QI_0_ measurements accounted for 43.9–80.9% of all data points, QI_1_ 16.4–54.8% and combined (QI_0_ + QI_1_) 88.6–98.9%. Within the QI_0_ and QI_1_ data, any singular observed values for heart rate ± 3 SDs of the mean value were removed as they were considered as extreme outliers, which resulted in < 5 data points being removed from approximate 12,000 QI_0_ and QI_1_ data points per biologger. No data cleaning was required for temperature measurements.

### Data Analysis

Weight, fork length, and condition factor (*K*) of tagged and untagged fish from the cage were compared using a series of two-sample *t*-tests. Tagged fish (*n* = 17) heart rate recovery and the re-establishment of diurnal rhythms are shown by graphing the hourly mean heart rate (±95% CI) over the first 12 days post-tagging and by calculating the daily (24 h) average heart rate. Differences in diurnal heart rate patterns amongst the three 14-day seasonal snapshots (winter, spring, and summer) are shown by graphing the hourly mean heart rate and temperature (± 95% CI) and analyzed using the daily (24 h) average heart rate, and the average day and night heart rates for each season. Day and night hourly heart rate measurements were split based on civil hours of darkness for mid Norway^2^. This meant for winter, starting on the 24th of February, night consisted of all the measurements taken from 17:00 to 08:00 for the first 7 days and then all the measurements from 18:00 to 07:00 for the next 7 days, ending on the 8th of March. For spring, starting on the 17th of April, night used all the measurements taken from 21:00 to 05:00 for all 14 days ending on the 30th of April. For summer, starting on the 2nd of June, night used all the measurements taken from 00:00 to 03:00 for all the 14 days, ending on the 15th of June. Amongst the seasons, the differences in heart rate [daily 24 h, night, day, and percentage change from day-to-day for daily (24 h) heart rate] were analyzed using non-parametric Friedman’s tests for non-independent samples. For crowding events, the average daily (24 h) heart rate and maximum hourly heart rate, before (pre), during (crowd), and after (post) crowding events (level 2, *n* = 17 fish; level 3, *n* = 6 fish) were analyzed using a non-parametric Friedman’s test for non-independent samples. *Post hoc* tests to compare significant differences between the seasons (winter, spring, and summer) or crowding days (pre, crowd, and post) were conducted using Wilcoxon signed-rank tests with the application of a Bonferroni correction. The strength of a linear relationship between heart rate and temperature was tested for each seasonal period using a Pearson product moment correlation. All analyses were conducted using SPSS 26 statistical software package. All error terms are ± 1 SEM (SE) unless stated otherwise.

## Results

### Tag Recapture

Of the 24 fish tagged and released into the cage, there were no recorded mortalities from the tagging date to harvest. At harvest, 5,865 fish were removed from the cage, indicating that the total mortality (excluding the fish taken for sampling, *n* = 90) for the cage population was 2.8% between the 3rd of February 2020 and the 2nd of July 2020. During the final sampling on the 22nd of June, 12 tagged fish were identified by their externally attached Floy tag near the dorsal fin. Further six fish were found at harvest on the slaughter boat on the 2nd of July, two were identified by their external Floy tag and the other four by remnant scar tissue between the pectoral fins from where the surgical incision was made during tag insertion. This resulted in a tag return rate of 18 from 24 (75%) (Table 1). No further tagged fish were retrieved or reported by staff in the processing plant, hence it is not known whether the remaining six tagged fish escaped from the cage, lost their tags, and died during the trial but were not found or were missed during harvest. For data analysis, 1 tag from the total of 18 recovered was excluded from the final data set as the tag was found dislodged inside the fish, which resulted in unusable heart rate data.

**TABLE 1:** Summary of fish recapture rate and tag retention, fork length, weight, and condition factor K of tagged fish at tagging and final.

**Heart**	**Length**	**Weight**	**Fish**	**Floy**	**Final**	**Final**	**Final**	**Sex**
**data**	**(mm)**	**(g)**	**re-captured**	**tag**	**length**	**Weight**	**K**	
				**retained**	**(mm)**	**(g)**		
Q	560	2,300	Y	Y	650	2,385	0.87	F
L	640	3,550	Y	Y	720	5,395	1.45	M
G	705	5,165	Y	Y	800	7,730	1.51	M
F	650	3,750	Y	Y	745	5,755	1.39	M
ND	620	3,300	Y	Y	690	4,105	1.25	F
A	655	4,265	Y	Y	760	5,930	1.35	M
B	610	3,075	Y	Y	735	4,600	1.16	F
I	625	3,620	Y	Y	750	5,779	1.37	F
O	660	4,505	Y	Y	770	6,220	1.36	M
K	610	3,200	Y	Y	685	4,355	1.35	F
E	575	3,120	Y	Y	670	4,455	1.48	F
J	625	3,370	Y	Y	730	5,445	1.40	M
C	590	3,185	Y*	Y	685	4,260	1.33	F
D	630	3,785	Y*	Y	730	5,570	1.43	F
M	630	3,120	Y*	N	–	–	–	–
N	655	3,820	Y*	N	–	–	–	–
H	635	3,590	Y*	N	–	–	–	–
P	640	3,685	Y*	N	–	–	–	–
ND	680	4,280	N	–	–	–	–	–
ND	645	3,990	N	–	–	–	–	–
ND	660	4,365	N	–	–	–	–	–
ND	655	4,320	N	–	–	–	–	–
ND	650	3,800	N	–	–	–	–	–
ND	575	2,640	N	–	–	–	–	–

(*) denotes tagged fish found on the slaughter boat, 10 days after the initial attempt to recapture tagged fish on the 22nd June 2020.

*Heart data letters (A–Q) refers to each fish with useable data in Figures 4, 5, ND indicates fish with no heart rate data.*

### Fish Length, Weight, and Condition Factor K

There was no initial difference in the condition between tagged and untagged fish (*t*-test, *df* = 52, *p* = 0.29, 0.12, and 0.53, for weight, fork length, and K, respectively, Table 2). During the final sampling on the 22nd of June, tagged fish (*n* = 12) were 20% lighter (weight: *t*-test, *df* = 40, *p* = 0.008) and 8% shorter (fork length: *t*-test, *df* = 40, *p* < 0.001) than untagged fish (*n* = 30) but still in the similar condition compared to untagged fish (*K*: *t*-test, *df* = 40, *p* = 0.35, Table 2).

**TABLE 2:** Size parameters (Mean ± 1 Standard Error) for weight (W), fork length (L_*F*_) and condition factor (K), of tagged and untagged fish in the sea cage in February (3rd to 11th) 2020 (at tagging) and at final sampling in June (22nd) 2020.

	**February 2020**			**June 2020**		
	**W (g)**	**L_*F*_ (cm)**	**K**	**W (g)**	**L_*F*_ (cm)**	**K**
Tagged	3,658 ± 130	63.3 ± 0.7	1.43 ± 0.02	5,142^a^ ± 335	72.3^a^ ± 4.2	1.34 ± 0.16
Untagged	3,366 ± 223	60.7 ± 1.5	1.41 ± 0.02	6,487^b^ ± 250	78.4^b^ ± 0.8	1.33 ± 0.02

*Different letters above mean values in June 2020 indicate a significant difference between tagged and untagged fish for that given parameter at *p* < 0.05.*

### Heart Rate Recovery Profile Post Tagging

**FIGURE 2:**
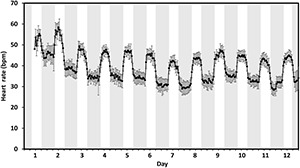
Post tagging recovery. The hourly mean heart rate measured in beats per min (bpm) of 17 fish tagged with DST centi-HRT tags between 11:00 and 14:00 (day 1) in early February 2020. Error bars represent ± 95% CI. Gray bands indicate approximate civil hours of darkness.

The highest hourly heart rates exceeded 55 bpm on the day of tagging and the 1st day of post-tagging, whilst a clear diurnal rhythm was established within 24 h of tagging (Figure 2). On day 4, heart rates had stabilized to within a range of 30–50 bpm with an average daily (24 h) heart rate of 39 ± 0.4 bpm (mean ± SE). On day 7, the average daily heart rate had dropped a further 3 bpm to 36 ± 0.4 bpm before increasing again to 38 ± 0.4 bpm on day 12. Average daily temperature over the 12 days was 7°C ± 0.1°C.

### Winter, Spring, and Summer Seasonal Trends

#### Heart Rate

**FIGURE 3:**
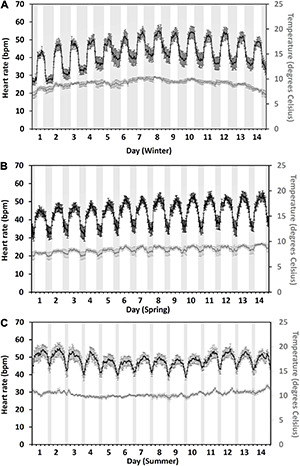
Fourteen-day, hourly mean heart rate (black) in bpm and temperature (gray) of 17 fish during winter **(A)**, spring **(B)**, and summer **(C)**. Gray bands indicate civil hours of darkness to the nearest hourly heart rate measurement (winter: the 24th of February ∼15 h, 17:00–08:00, decreasing to ∼13 h by the 8th of March, 18:00–07:00; spring: the 17th to the 30th of April ∼8 h, 21:00–05:00; summer: the 2nd to the 15th of June ∼3 h, 00:00–03:00. Error bars represent ± 95% CI.

Diurnal heart rate patterns during each 14-day seasonal period closely followed the seasonal day/night cycle with lower heart rates observed at night and higher during the daytime (Figure 3). During winter (Figure 3A), the average daytime heart rate (50 ± 0.3 bpm, mean ± SE) was 11 bpm, which was higher than the night heart rate (39 ± 0.2 bpm). In spring (Figure 3B), the daytime heart rate (48 ± 0.2 bpm) was 11 bpm, which is higher than the night (37 ± 0.2 bpm). In summer (Figure 3C), the daytime heart rate (49 ± 0.2 bpm) was 6 bpm, which is higher than the night (43 ± 0.3 bpm). Average daily (24 h) heart rates were 44 ± 0.9 bpm in winter, 43 ± 0.6 bpm in spring, and 46 ± 0.5 bpm in summer.

Across the seasons, there was a difference in the daily (24 h) heart rate [season daily: χ^2^_(2)_ = 13.8, *p* = 0.01]. *Post hoc* analysis using a Bonferroni corrected *p* < 0.017 showed that fish had 8% higher heart rates in summer compared to spring but showed no differences to winter (summer vs. spring, *Z* = −3.1, *p* = 0.001; summer vs. winter, *Z* = 1, *p* = 0.3; spring vs. winter, *Z* = −2.1, *p* = 0.04). Whilst the percent change in the average daily heart rate from 1 day to the next was the same for all the seasons [season percent change: χ^2^_(2)_ = 4.7, *p* = 0.1] and was 3.6% ± 0.7% in winter, 1.9% ± 0.4% in spring, and 2.2% ± 0.5% during summer.

The difference in daily (24 h) heart rate across the seasons was due to differences in night heart rates [season night: χ^2^_(2)_ = 13, *p* = 0.002], not daytime [season day: χ^2^_(2)_ = 5.3, *p* = 0.07]. *Post hoc* analysis of night heart rates using a Bonferroni corrected *p* < 0.017 indicated that fish have 14% higher night heart rates in summer compared to spring but not being different from winter (summer vs. spring, *Z* = 3.3, *p* = 0.001; summer vs. winter, *Z* = 2.2, *p* = 0.3; spring vs. winter, *Z* = −1.7, *p* = 0.08).

#### Temperature

Water temperatures experienced by fish followed a similar diurnal pattern to that of heart rate (Figure 3) and were most strongly correlated with heart rate during the spring 14-day period (spring heart rate: *y* = 8.78 × Temp–29.3, *r* = 0.82, *p* < 0.001; Winter heart rate: *y* = 5.5 × Temp–6.86, *r* = 0.56, *p* < 0.001; Summer heart rate: *y* = 3.21 × Temp–15.1, *r* = 0.46, *p* < 0.001). The average daily temperature during the 14-day winter period was 9.1°C ± 0.2°C (mean ± SE), in spring 8.3°C ± 0.2°C, and in summer 10.3°C ± 0.1°C. In winter, the highest daily (24 h) temperature experienced by tagged fish (10°C ± 0.2°C) occurred on day 8 (Figure 3A) and corresponded with the highest daily (24 h) heart rate of 49 ± 2.3 bpm. The same occurred for spring with the highest daily temperature (9.1°C ± 0.3°C) on day 14 (Figure 3B) aligning with the highest daily heart rate of 47 ± 2.1 bpm. However, for summer the highest daily temperature (11.1°C ± 0.2°C) on day 14 (Figure 3C) did not correspond to the highest daily heart rate (51 ± 2.1 bpm), which occurred on day 2 (Figure 3C). Day 2 summer daily temperature averaged 10.5°C ± 0.2°C, and the day 14 daily heart rate was 49 ± 1.5 bpm.

### Effects of Level 2 and 3 Crowding Events

#### Level 2 Crowding

**FIGURE 4:**
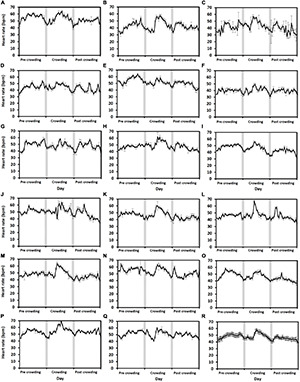
Level 2 crowding event fish response. Average hourly heart rate (black line) in bpm of 17 individual salmon [Graphs **(A–Q)**] undergoing a level 2 crowding event. Hourly heart rates are shown from the day prior to crowding (the 15th of June), on the day of crowding (the 16th of June), and the day post-crowding (the 17th of June). Error bars for individual fish graphs represent hourly mean heart rate ± 95% CI. Graph **(R)** represents the average hourly heart rate of all 17 fish (Error bars show ± 95% CI). Gray bars indicate approximate civil hours of darkness.

For the level 2 crowding event, the highest hourly heart rates varied by > 20 bpm amongst fish (*n* = 17) on the day of crowding (the 16th of June, Figures 4A–Q). The fish with the greatest response to crowding had the highest hourly heart rate of 70 ± 1.1 bpm (mean ± 95% CI, Figure 4L) and the fish exhibiting the least response 49 ± 2.1 bpm (Figure 4F). Across all the fish (Figure 4R), the highest hourly heart rates differed over the 3 days [χ^2^_(2)_ = 22.7, *p* < 0.001]. *Post hoc* analysis using a Bonferroni corrected *p* < 0.017 found that the highest hourly heart rates were higher on the day of crowding (60 ± 0.6 bpm, mean ± SE) compared to pre-crowding (55 ± 0.6 bpm) and post-crowding (53 ± 0.6 bpm) days (pre vs. crowd: *Z* = 2.68, post vs. crowd: 3.62, *p* = 0.007 and *p* < 0.001, respectively), whilst there was no difference between pre- and post-crowding days (pre vs. post: *Z* = −2.29, *p* = 0.02). A different pattern across days was found when comparing daily (24 h) heart rates [χ^2^_(2)_ = 22.7, *p* < 0.001] as there was no difference between the pre-crowding (49 ± 1.5 bpm) and crowding day (49 ± 1.6 bpm, pre vs. crowd: *Z* = 0.3, *p* = 0.7) whilst the post-crowding daily heart rate (45 ± 1.4 bpm) was approximately 8% lower than both (pre vs. post: *Z* = −3.6, crowd vs. post: 3.6, *p* < 0.001 for both).

#### Level 3 Crowding

**FIGURE 5:**
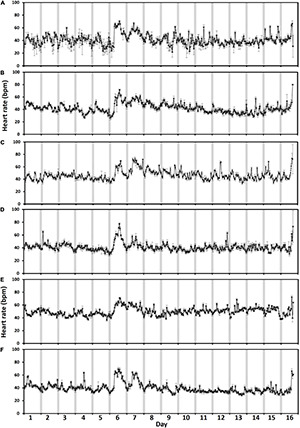
**(A–F)** Individual fish response to an intensive sample event. Individual hourly heart rate in beats per minute (bpm) of the six six salmon (Fish C, D, H, M, N and P from Table 1) collected at harvest. Heart rate is shown from the 5 days prior to final cage sampling (days 1–5, the 17th to the 21st of June), fish sampling from the cage (day 6, the 22nd of June and post-sampling till harvest (days 7–16, the 23rd of June to the 2nd of July). Error bars represent ± 95% CI. Gray bars indicate approximate hours of civil darkness.

**FIGURE 6:**
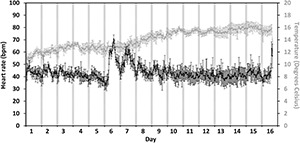
Average fish response to a level 3 crowding event. Average hourly heart rate (black line) in bpm and temperature experienced (gray line) of six salmon collected at harvest. Heart rate and temperature are shown from the 5 days prior to final cage sampling (days 1–5, the 17th to the 21st of June), intense fish sampling (level 3 crowding event) from the cage (day 6, the 22nd of June) and post sampling till harvest (days 7–16, the 23rd of June to the 2nd of July). Error bars represent ± 95% CI. Gray bars indicate approximate civil hours of darkness.

For the level 3 crowding event, the highest hourly heart rates varied by > 15 bpm amongst fish (*n* = 6) on the day of crowding (the 22nd of June, Figure 5, day 6). The fish that showed the greatest response to crowding had the highest hourly heart rate of 78 ± 1.4 bpm (mean ± 95% CI, Figure 5M) whilst the fish showed the least response of 70 ± 4.1 bpm (Figure 5P). Across all fish (Figure 6), there was a difference amongst days for the highest hourly heart rate [χ^2^_(2)_ = 10.3, *p* = 0.006]. The trend indicated higher hourly heart rates on both the day of crowding (72 ± 1 bpm, mean ± SE, Figure 6, day 6) and post-crowding (65 ± 1.7 bpm, Figure 6, day 7) compared to pre-crowding (48 ± 1.6 bpm, Figure 6, day 5), but *post hoc* analysis (using a Bonferroni corrected *p* < 0.017) did not statistically differentiate amongst days (pre vs. crowd, *Z* = 2.2, *p* = 0.03; pre vs. post, *Z* = 2.2, *p* = 0.03, crowd vs. post, *Z* = − 1.8, *p* = 0.08). The same pattern amongst days occurred for daily (24 h) heart rates [χ^2^_(2)_ = 9, *p* = 0.01] with the trend indicating ∼30% higher daily heart rates on both the days of crowding (53 ± 2.3 bpm) and post-crowding (53 ± 4.2 bpm) compared to pre-crowding (39 ± 2.8 bpm), but *post hoc* analysis (using a Bonferroni corrected *p* < 0.017) did not statistically differentiate between days (pre vs. crowd, *Z* = 2.2, *p* = 0.03; pre vs. post, *Z* = 2.2, *p* = 0.03, crowd vs. post, *Z* = 0.1, *p* = 0.9). At crowding for harvest (Figures 5, 6, day 16), whilst no analysis was performed due to uncertainties in the timing of pumping and fish slaughter procedures, all six fish began to display increases in heart rate similar to the level 3 crowding event.

## Discussion

Here, we have demonstrated that DST centi-HRT biologgers can be used for long-term monitoring of heart rates in farmed Atlantic salmon in a commercial scale sea cage as 17 out of the 18 biologgers retrieved the produced useable data across the entire trial period. After implanting, tagged fish appeared to recovery quickly in terms of heart rate pattern; however, they were 20% lighter at harvest, indicating that the biologgers are still impeding tagged fish welfare in some ways. Over a 5-month trial, heart rate patterns closely followed a day/night diurnal cycle with fish exhibiting heart rates at night that were between 6 and 11 bpm lower than during the daytime. The most noticeable change over the trial was that average night time heart rate levels increased during the summer season, whilst average daytime heart rates changed very little across the seasons. Crowding tests highlighted that individual fish heart rate response is highly variable during short (<3 h) level 2 crowding events, but all appear to recover within 24 h, whilst all fish will show a noticeable response to a level 3 (>5 h) crowding event and heart rate recovery takes longer than 24 h.

### Fish Recovery and Growth

Fish recovery in terms of normalized heart rate pattern after tagging can range from days to weeks in tank trials (Hvas et al., 2020b; Føre et al., 2021; Zrini and Gamperl, 2021), and a regular diurnal heart rate cycle is often described as an indicator of a normal fish behavior and hence used as a sign fish have recovered from a tagging procedure (e.g., Brijs et al., 2019b; Føre et al., 2021). However, there is often a concern that the process of adding a tag, or the tag itself, will alter a fish’s physiology so that it is no longer representative of untagged fish (Cooke et al., 2011; Jepsen et al., 2015; Macaulay et al., 2021). In our trial, tagged fish established a diurnal rhythm within 24 h of having the bio-loggers implanted and mean daily heart rates stabilized around 39 bpm in ∼7°C water temperature within 4 days, indicating in terms of heart rate pattern, these fish recovered from tagging very quickly. However, the 20% reduced growth rate in tagged fish suggests otherwise and indicates that a fish’s heart rate pattern alone in a sea cage environment is not a fail proof indication that a fish has fully recovered from being tagged. Furthermore, whilst no tagged fish mortalities were observed during the 5-month trial period, six fish were never found, meaning it is unknown, if these six fish did or did not, initially recover from being tagged. Unexplained tagged fish loss is an ongoing issue with long-term tagging studies (Macaulay et al., 2021) that is still yet to be resolved.

Whilst the data from sea cage field studies on the effects on growth in fish tagged with heart rate biologgers are lacking (e.g., Gamperl et al., 2021), a similar reduction in growth seen in our study also occurred in a 12-week tank trial (Hvas et al., 2020a). Reduced growth may be due to the tagging process resulting in a limited growth rate for a period whilst fish are recovering, and hence simply do not catch up to untagged fish in terms of lost growth. The finding here, that there was no difference in fish condition between tagged and untagged fish, may support this theory. However, a recent study on compensatory growth rates in farmed salmon that experienced 8 weeks of fasting (Hvas et al., 2021) indicates that if the effect of tagging was only short term, tag fish should still have regained the lost growth by harvest time. If future studies compared daily growth rings in the otoliths (Wright et al., 1991) or scales (Fukuwaka, 1998) between tagged and untagged fish, this may pinpoint when differences in growth occurred to confirm whether in a sea cage, it is a short-term effect on the fish whilst recovering from tagging, or rather a long-term effect of fish having to live with an internal tag.

### Seasonal Heart Rate Patterns

Over a 5-month trial, heart rates in farmed Atlantic salmon followed a strong seasonal diurnal pattern that overlapped with the day/night cycle, and that these patterns were also strongly correlated with a fish temperature experience, particularly in winter and spring (Figures 3A,B). These observations are consistent with previous research, which has found that many species of fish often demonstrate circadian rhythms, for example, brown trout (*Salmo trutta*, Priede and Young, 1977), Mediterranean seabream (*Sparus aurata*, Aissaoui et al., 2000), and Atlantic salmon (*Salmo salar*, Gamperl et al., 2021) which can be closely linked to the day/night light cycles (Aissaoui et al., 2000; Gamperl et al., 2021).

In addition to seasonal changes in a diurnal pattern, daily (24 h) heart rates were higher in summer than spring due to higher night time heart rate levels, whilst there was little difference in daytime heart rate levels across all seasons. Slight differences in the day/night temperature range in spring (∼2°C, Figure 3B) compared to summer (∼1°C, Figure 3C) may partially account for the difference seen between spring and summer day/night heart rates (spring day/night difference was 11 bpm, summer 6 bpm). However, higher average heart rates observed at night in summer may also be due to the shorter period of darkness (∼3 h) limiting the fish’s ability to reduce their activity (i.e., rest). Salmon typically reduce their activity at night through lowered swim speeds compared to the daytime (e.g., Oppedal et al., 2011; Hansen et al., 2017). Hence, during summer, if fish are spending most of their time being active (up to 21 h light per day), they may not fully reduce their activity levels. The average night time heart rate measured in summer of 43 bpm is also well above the estimated resting heart rates for salmon (∼25–39 bpm, Hvas et al., 2020a; Zrini and Gamperl, 2021) and hence further supports the concept that salmon in sea cages in summer may never fully rest. However, Gamperl et al.’s (2021) field study on Atlantic salmon during a summer heatwave concluded, that the differences they found between in day/night heart rate levels were mainly due to the fish’s circadian rhythm, and not differences in fish activity levels between day and night. Further studies on salmon in a sea cage environment are needed to expand our knowledge around farmed fish heart beat variation between day and night and whether it can be used as an indicator of stress or detrimental changes in fish behavior.

### Crowding Events

Disturbance events in a sea cage, such as crowding, can increase fish activity, resulting in increased stress levels in salmon that can be detectable by an increase in heart rate (Svendsen et al., 2021). In a controlled tank environment, stressing fish by crowding them for 30 min elevated heart rates substantially, however, fish recovered within 24 h (Hvas et al., 2020a). Furthermore, consecutive stress events like crowding plus netting, may cause heart rates to stay elevated for longer periods (Brijs et al., 2019a). In our trial, the level 2 crowding event found that some fish showed a noticeable heart rate response (e.g., Figure 4L) whilst other fish had no obvious response (e.g., Figure 4F). Furthermore, whilst on average the highest hourly heart rates occurred on the day of crowding indicating an increase in stress, the daily (24 h) average heart rate on the day of crowding was no different to the precrowding day. Hence, at the cage population level, if the level 2 crowding event was not known to have occurred, based on the biologger heart rate responses, it may have been difficult to determine whether fish had endured a stress event, particularly if only using the average daily (24 h) heart rate as an indicator.

For the second crowding event, fish experiencing the equivalent of a level 3 crowding, which is categorized as “undesirable” (Noble et al., 2018). Individually, all the fish showed a noticeably response in terms of having elevated heart rates on the crowding day and post-crowding day (Figure 5). In addition, on the day of crowding, the daily (24 h) average heart rate was 30% higher than the pre-crowding day, which is a stark difference to the typical change in average daily heart rates (2–4%) measured across all the three seasonal periods. On the day of post-crowding, the daily (24 h) heart rate was still 30% higher than the pre-crowding day, further indicating that at a population level, the level 3 crowding event has a longer lasting (>24 h) influence on fish stress. The highest hourly heart rates measured during the level 3 event are still below the maximum heart rates of 80 and 100 bpm estimated in laboratory studies on Atlantic salmon (Lucas, 1994; Hvas et al., 2020a; Zrini and Gamperl, 2021), which suggests that, whilst categorized as undesirable crowding, it is unlikely that the level 3 crowding event in this study lasted long enough to forced fish to reach their maximum heart rates or maximum stress levels.

These crowding test results demonstrate that heart rate biologgers could be used to monitor sudden increases in stress levels of a fish population in a sea cage and indicate the time required for fish to recover. In terms of practical application, detecting meaningful changes in fish heart rate in a sea cage will require the assessment of heart rate patterns in multiple ways, to gain a better understanding of how stressful an event may have been. For example, for the level 2 crowding event, the change in the highest hourly heart rate on the day of crowding gave an indication that a stress event occurred, whereas the change in daily (24 h) heart rate gave no indication. Whereas, for the level 3 event, the hourly highest heart rate and the change in daily (24 h) heart rate both indicated that a stress event has occurred and that its effects are felt by the fish for longer than 24 h. The individual response of sentinel fish in a cage could also be used to assess the effect size of a stress inducing event, rather than a simple yes/no answer, particularly if it is relatively moderate (e.g., levels 1–2 crowding). For example, as with the level 2 event herein, only some fish showed a stress response, hence the proportion of sentential fish in a cage that show a response could be extrapolated out to what proportion of the cage population may have been stressed by an event.

One of the main limitations of using DST biologgers in terms of practical application in sea cages is that the data are only captured and analyzed after the loggers have been removed from the fish. Whilst this is okay at experimental and exploratory stages for gaining and an understanding of typical heart rate patterns of farmed fish, in the future real-time access to the data will be more beneficial. For example, if an acoustic telemetry version of a heart rate biologger was developed to enable real-time data capture, this could be highly valuable to farmers, in that the stress levels of fish could be closely monitored in the days before and after a crowding, delousing, extreme weather conditions, or other major handling events. Management decision-making could then be “fact-based” guided, to reduce multiple stressors, aid in securing welfare, and lower the likelihood of mass mortalities occurring during major disturbances.

## Conclusion

We have shown that it is possible to use heart rate biologgers to monitor seasonal diurnal rhythms, and assess the effects of level 2 and 3 crowding events on fish stress and recovery times in a commercial scale sea cage. While physiological data taken from fish with internal tags do need to be treated with caution (Macaulay et al., 2021) and that here, tagged fish were growth compromised, they displayed clear diurnal heart rate rhythms over a 5-month trial. This indicates that as sentinel monitors of fish welfare, the tagged fish were reasonably representative of the cage fish population. Future studies using heart rate biologgers in fish that investigate combinations of potential environmental stresses, for example, low oxygen environments (Remen et al., 2013) or submergence (Oppedal et al., 2020) with crowding and delousing events, should be approached with caution as under multiple stressor environments (e.g., Wright et al., 2019) it will be essential to determine whether the influence of sublethal effects occurring in fish with tags does not influence the data collected to such an extent, that these fish become unrepresentative of the sea cage population.

## Data Availability Statement

The original contributions presented in the study are included in the article/supplementary material, further inquiries can be directed to the corresponding author/s.

## Ethics Statement

The animal study was reviewed and approved by Norwegian Animal Research Authority (permit no. 19629).

## Author Contributions

FW-M programmed tags, tagged fish, processed and analyzed tag data, conceptualized, and wrote first full draft of the manuscript. MH provided intellectual guidance on tag use and fish welfare during tagging procedure, commented on all drafts and co-wrote a later draft of the manuscript. TV monitored fish during the 5 month trial and assisted with simulated crowding events, re-captured the tagged fish, downloaded raw data, and commented on final draft of the manuscript. TD provided conceptual design for tagging trial and data collection. FO provided conceptual design input, directed simulated crowding events, and commented on all the manuscript drafts. All authors contributed to the article and approved the submitted version.

## Conflict of Interest

The authors declare that the research was conducted in the absence of any commercial or financial relationships that could be construed as a potential conflict of interest.

## Publisher’s Note

All claims expressed in this article are solely those of the authors and do not necessarily represent those of their affiliated organizations, or those of the publisher, the editors and the reviewers. Any product that may be evaluated in this article, or claim that may be made by its manufacturer, is not guaranteed or endorsed by the publisher.

## Footnotes

1
www.star-oddi.com

2
www.timeanddate.com
